# All Patients with ADPKD Should Undergo Screening for Intracranial Aneurysms: COMMENTARY

**DOI:** 10.34067/KID.0000000000000328

**Published:** 2023-11-22

**Authors:** Arlene B. Chapman

**Affiliations:** University of Chicago, Section of Nephrology, Department of Medicine, Chicago, Illinois

**Keywords:** ADPKD

In this issue of Kidney360, Park *et al.* and Chebib *et al.* provide extensive and strong rationales regarding the value and the concern for screening all patients with autosomal dominant polycystic kidney disease (ADPKD) for the presence of intracranial aneurysms (ICAs). Rupture of an ICA is a devastating complication of ADPKD which all patients worry about. Those who have experienced a ruptured ICA in their family carry this anniversary forever, and it is a part of every clinic visit they have for ADPKD. Most patients with ADPKD who have researched their condition typically ask this question first to their providers: Do I have an ICA, or should I be screened for an ICA? Thankfully, some of the level of concern for having an ICA has receded in recent years because the estimated frequency of ICA in ADPKD has dropped considerably from 40% in the original small retrospective case series that included symptomatic ruptures to somewhere between 5% and 8% in larger series of asymptomatic individuals with ADPKD prospectively imaged.^1^

How best to reliably identify those who will develop an ICA is not yet determined. Neither genetics nor blood nor urine biomarkers help us. General demographics by age, female sex, and known risk factors for ICA such as smoking and hypertension help a little.^1–3^ In addition, now newly defined impact with regard to the value of screening for ICA is available. True that today it is easier, less expensive, and more reliable to noninvasively screen for ICA using magnetic resonance angiography. However, uncertainty remains. Yes, ICA found in patients with ADPKD are generally smaller and do not typically grow as fast as ICA in the general population, but yes, ICA tend to rupture at a smaller diameter in patients with ADPKD compared with the general population.^2,3^

Data now demonstrate a measurable NEGATIVE impact of diagnosing an ICA in an asymptomatic individual. Many times, with small ICA, the appropriate approach is to observe over time as ICA in patients with ADPKD tend to grow slowly if at all and may never rupture. This then leads to increasing anxiety and depression and social isolation. In addition, elective radiological or surgical intervention can associate with complication rates greater than the expected rate of rupture.^4^

What to do then? Some recommendations are relatively clear: Recommend screening those with a positive family history of ICA, particularly those with a family history of a ruptured ICA and recommend screening those who have professions that would put others at risk, for example airline pilots. Potentially consider recommending screening those who are preparing for the precious gift of a kidney transplant or who will be undergoing complicated large surgeries. The rest? Everyone could be offered screening for ICA, but patients could choose not to undergo screening. It really is on a case-by-case basis. With this approach, as in genetic counseling, pretest counseling is key. How will they feel if a small ICA is found that would not be intervened on? How would they feel if surgical intervention with potential complications that are life altering would be recommended? They may decide not to move forward with screening and take on the risk of a ruptured ICA. We need more data about predictive biomarkers for those ICA that will rupture to help us refine our choices. And of course, we need time to talk about these very important issues with our patients.
